# Combined fluorescent seed selection and multiplex CRISPR/Cas9 assembly for fast generation of multiple *Arabidopsis* mutants

**DOI:** 10.1186/s13007-021-00811-9

**Published:** 2021-10-30

**Authors:** Robertas Ursache, Satoshi Fujita, Valérie Dénervaud Tendon, Niko Geldner

**Affiliations:** 1 Department of Plant Molecular Biology, University of Lausanne, 1015 Lausanne, Switzerland; 2 Present Address: UMR5546 CNRS, Toulouse-INP, University of Toulouse, 24 Chemin de Borde Rouge, Auzeville Tolosane, 31320 France

**Keywords:** Plant gene editing, Fluorescent seed selection, Co-transformation, *Cas9*, *zCas9i*, *pEC1*.2, *PcUBi4*-2

## Abstract

**Background:**

Multiplex CRISPR-Cas9-based genome editing is an efficient method for targeted disruption of gene function in plants. Use of CRISPR-Cas9 has increased rapidly in recent years and is becoming a routine method for generating single and higher order *Arabidopsis thaliana* mutants. Low entry, reliable assembly of CRISPR/Cas9 vectors and efficient mutagenesis is necessary to enable a maximum of researchers to break through the genetic redundancy within plant multi-gene families and allow for a plethora of gene function studies that have been previously unachievable. It will also allow routine de novo generation of mutations in ever more complex genetic backgrounds that make introgression of pre-existing alleles highly cumbersome.

**Results:**

To facilitate rapid and efficient use of CRISPR/Cas9 for *Arabidopsis *research, we developed a CRISPR/Cas9-based toolbox for generating mutations at multiple genomic loci, using two-color fluorescent seed selection. In our system, up-to eight gRNAs can be routinely introduced into a binary vector carrying either a FastRed, FastGreen or FastCyan fluorescent seed selection cassette. FastRed and FastGreen binary vectors can be co-transformed as a cocktail via floral dip to introduce sixteen gRNAs at the same time. The seeds can be screened either for red or green fluorescence, or for the presence of both colors. Importantly, in the second generation after transformation, *Cas9* free plants are identified simply by screening the non-fluorescent seeds. Our collection of binary vectors allows to choose between two widely-used promoters to drive Cas enzymes, either the egg cell-specific (*pEC1.2*) from *A. thaliana* or the constitutive promoter from *Petroselinum crispum* (*PcUBi4-2*). Available enzymes are “classical” *Cas9* codon-optimized for *A. thaliana* and a recently reported, intron-containing version of *Cas9* codon-optimized for *Zea mays*, *zCas9i*. We observed the highest efficiency in producing knockout phenotypes by using intron-containing *zCas9i* driven under egg-cell specific *pEC1.2* promoter. Finally, we introduced convenient restriction sites flanking promoter, *Cas9* and fluorescent selection cassette in some of the T-DNA vectors, thus allowing straightforward swapping of all three elements for further adaptation and improvement of the system.

**Conclusion:**

A rapid, simple and flexible CISPR/*Cas9* cloning system was established that allows assembly of multi-guide RNA constructs in a robust and reproducible fashion, by avoiding generation of very big constructs. The system enables a flexible, fast and efficient screening of single or higher order *A. thaliana* mutants.

**Supplementary Information:**

The online version contains supplementary material available at 10.1186/s13007-021-00811-9.

## Background

Generating targeted genetic changes in living cells and organisms has historically been a great challenge in many species, including plants. Precise editing and regulation of genomic information is essential for understanding gene function, production of new plant traits and developing new plant breeding strategies. During the past decade, technological breakthroughs have finally enabled plant genome editing [1–3]. The latest breakthrough was achieved with the discovery of CRISPR/Cas-based systems, a gene editing technology which allows us to knock genes in or out [4–7]. Knocking out a gene in plants involves expressing CRISPR/Cas and directing it to a specific genomic locus using a guide RNA. There, Cas protein induces sequence-specific DNA double-strand breaks (DSBs), and the cell’s DNA repair mechanism fixes the cut using non-homologous end joining (NHEJ) or homology-directed recombination (HDR). NHEJ occurs most often, is highly efficient but inaccurate, and tends to introduce errors in the form of small insertions or deletions that are usually sufficient to knock out the gene. This technique is widely used to produce stable single and multiple mutants in various plant species. It is a very valuable tool in studying gene function, breaking the redundancy in multigene families and developing new plant traits [4, 8–12]. Although multiple Cas proteins have been tested for gene editing in plants, *Cas9* and *Cpf1* (now known as* Cas12a*) are currently most widely used based on the nature of their interference complex and their efficiency [9, 13–17]. CRISPR/Cas9 consists of two main components: the Cas nuclease and a guide RNA (gRNA). The gRNA is made up of a single guide RNA (sgRNA), a short, 17–20 nucleotide sequence complementary to the target genomic DNA, and a tracr RNA (trans-activating crRNA), which serves as a binding scaffold for the Cas nuclease. The sgRNA and Cas protein form a Cas/sgRNA complex which is guided to a specific genomic locus site using Watson–Crick base pairing. This results in the cleavage of target DNA sequences adjacent to the PAM (protospacer-adjacent motif), a short, few-nucleotide-long sequence that is crucial for Cas binding [18, 19]. Recently, Grützner et al. [20] reported that the addition of 13 introns into the *Cas9* coding sequence in combination with two nuclear localization signals (NLS) led to higher accumulation of the *Cas9* in the nucleus and significant improvement of editing efficiency.

In plants, two types of *Agrobacterium*-mediated techniques are used to create transgenic lines carrying the CRISPR/Cas system: in planta transformation and callus-based transformation [21–25]. The most typical example of in planta transformation is floral dip-based, where *Arabidopsis *egg cells have been suggested to be the target of the T-DNA transferred by *Agrobacterium* infection [21, 24, 26]. In case of callus-based systems, excised or partially disrupted meristems are transformed, subjected to antibiotic or herbicide selection, and then carried through tissue culture to regenerate shoots and roots from the transformed tissues [27–29]. *Arabidopsis *is highly amenable to in planta transformation, giving it an additional advantage as a simple and efficient model organism, compared to many other plant species where in planta transformation methods have often failed and which have to rely on, work- and time-intensive tissue culture protocols.

In theory, the CRISPR/Cas system should be able to function and induce mutations in egg cells or zygotes. However, several studies have demonstrated that the occurrence of such early, complete mutations in the first generation is rare in *Arabidopsis *and depends on the strength and specificity of promoters driving the Cas expression. When driven under ubiquitous promoters, such as Cauliflower Mosaic Virus 35S promoter (CaMV 35S) or parsley *PcUBi4-2*, a large number of editing events occur later in development, generating a high degree of mosaicism in plants of the first generation (T1) and the detection of mutations that are not necessarily transmitted to the next generation. This indicates that CRISPR/Cas9-induced mutations in *Arabidopsis *occur mostly after the first embryonic cell division, at various stages of plant development [13, 30–32]. The use of more specific promoters to drive the expression of Cas enzymes in Arabidopsis, such as egg cell-specific *pEC1.2*, egg cell and early embryo-specific *pDD45*, or cell division-specific *pYAO* promoter, led to a more efficient generation of non-mosaic, biallelic mutants for multiple target genes in *Arabidopsis *in the T1 generation [31–34].

Here, we developed a toolbox with a straightforward cloning protocol for assembly of multiple gRNAs together into transfer DNA (T-DNA) vectors for *Agrobacterium*-mediated transformation into Arabidopsis. The assembly is based on an efficient combination of simple Golden Gate and Gateway cloning methods, allowing an average molecular biologist to reproducibly assemble the constructs of interest. The T-DNA transfer vectors contain FastRed, FastGreen or FastCyan fluorescent seed selection markers for a fast seed selection under fluorescent stereomicroscopes. We observed that FastRed and FastGreen cassette containing vectors can be easily co-transformed together as an *Agrobacterium* cocktail to screen for the presence of both seed selection markers in *Arabidopsis *seeds. This allows to double the amount of target sgRNAs, while also allowing to avoid generation of very large constructs that are difficult-to-assemble and possibly unstable. Importantly, it also enables screening for different combinations of mutants in the same generation. The fluorescent seeds can be screened fast and efficiently for successful transformation events, as well for editing events already in T1 generation avoiding the antibiotic-caused stress and plant growth defects. In the T2 generation, *Cas9*-free plants carrying the desired homozygous events in multiple genes can be easily identified by counterselection of non-fluorescent seeds. Although we use *Cas9* and intronized *zCas9i* from *Streptococcus pyogenes* driven from *PcUBi4-2* and *pEC1.2* as examples in this study, we expanded our multicolor T-DNA toolbox by introducing the *SaCas9* from *Staphylococcus aureus* and *AsCas12a* from *Acidaminococcus* into our T-DNA vectors for further system modifications. Both, *SaCas9* and *AsCas12a*, have different PAM specificities, allowing to choose from a broader pool of sgRNAs [13, 35]. At the same time, *SaCas9* requires longer PAMs compared to Sp*Cas9*, which should significantly reduce the frequency of off-targets [13]. The PAM requirement for *AsCas12a*, on the other hand, is “TTTN” which favors its use in targeting AT-rich genomic regions and has been successively used to generate targeted mutations as well as achieve transcriptional regulation in different organisms. Moreover, differently from CRISPR-Cas9 systems, *AsCas12a* does not require tracrRNA and RNase III for processing of mature crRNA. The transformation of pre-crRNA to mature crRNA is mediated by intrinsic ribonuclease activities of* Cas12a* domains [35]. Finally, to expand the possibilities for future modification of some of our T-DNA vectors, we introduced convenient restriction sites flanking the promoter, *Cas9* and the fluorescent seed selection cassette, to be able to select any promoter, Cas protein or selection cassette of interest. This adds an important element of modularity to our system that is absent in many other current systems.

## Results

### An efficient assembly system for multiplexed CRISPR/Cas9

We sought to design an efficient and easy-to-use CRISPR/Cas9 system for the plant research community. Potential applications of such system would include, but are not limited to: (1) simultaneous targeted mutagenesis at multiple *Arabidopsis *genomic loci; (2) generation of targeted large deletions; (3) further modification of single-site Gateway-based T-DNA vectors by introducing newly discovered Cas proteins, more efficient promoters to drive Cas expression or desired selection cassette.

**Fig. 1:**
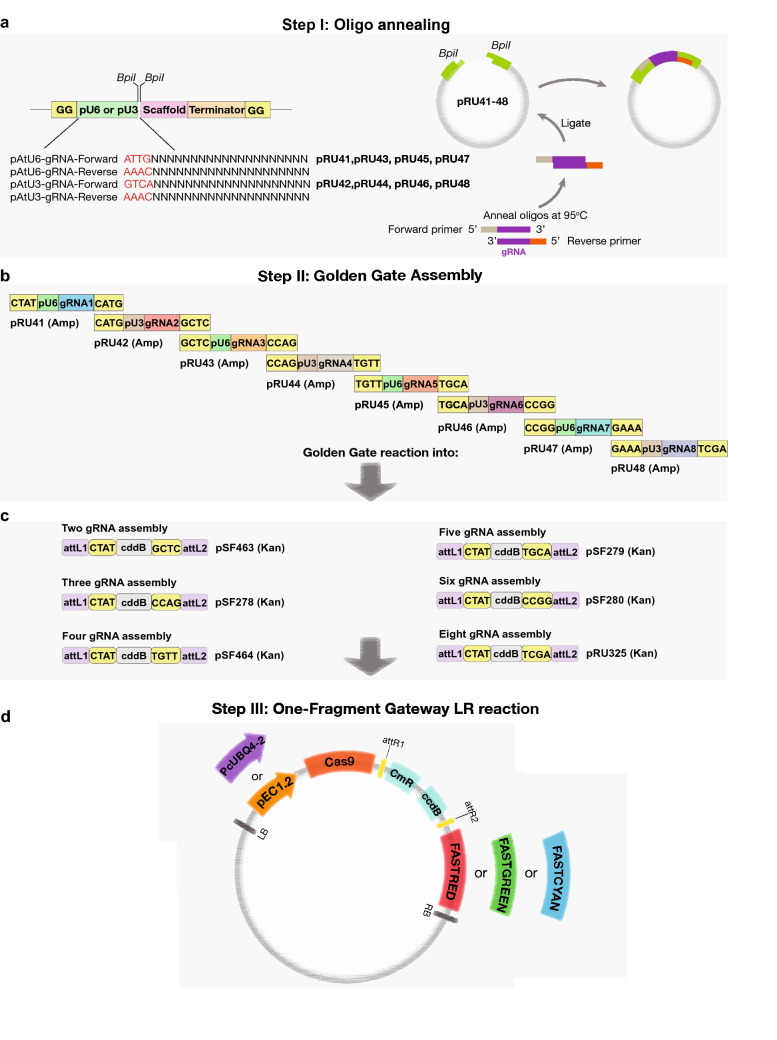
CRISPR/Cas cloning strategy. **a** Oligo annealing-based cloning of chosen gRNAs into pRU41-48 entry vectors containing *pU6* and *pU3* promoters. **b**, **c** Golden Gate assembly of up to eight gRNAs into corresponding intermediate vectors containing attL1-attL2 for single Gateway LR reaction. **d** Final single Gateway assembly into T-DNA vectors containing *PcUBi4-2* or *pEC1.2* promoters driving *Cas9* of interest. The vectors contain one of three fluorescent seed selection cassettes, FastRed, FastGreen and FastCyan

We therefore developed a system that allows for a reliable and routine assembly of multiple gRNAs into a T-DNA destination vector already containing *Cas9*. The assembly method combines both, Golden Gate assembly and Single Gateway-based recombination (Fig. 1). Three steps are required for assembly. The first step includes generation of entry clones where sgRNAs are introduced into a vector containing either the *pU6* or *pU3* promoter and gRNA scaffold via a simple oligo annealing technique. This is a single tube reaction and only requires an annealed oligonucleotide pair to serve as the sgRNA molecule of choice to be introduced into a convenient *BbsI* restriction site (Fig. 1a; Additional file 1: Table S1). We chose the oligo annealing technique for introducing 20 nt sgRNAs into *BbsI* containing entry clones based on its previous, highly successful application [13, 30, 32, 36–39]. These gRNA entry clones contain overhangs to enable a one-step Golden Gate assembly. This step of cloning relies on *BsaI* which belongs to the Type IIS restriction enzyme family that cleave outside their respective recognition sequences (Fig. 1b, c; Additional file 1: Table S2) [40]. We combined the efficiency and versatility of the Golden Gate cloning approach with another method that allows sequence-independent cloning, namely single-fragment Gateway cloning. This approach relies on the recombination between att sites mediated via a commercially available enzyme set, LR clonase (Thermo Scientific). Gateway cloning is used in many fields and widely adopted vector collections have been developed [6, 41–47]. The Gateway cloning has been successfully used to generate multiplexed CRISPR-Cas9 systems for plant genome editing [48–52]. Therefore, the set of Golden Gate recipient vectors generated in this study contain the ccdB counterselection cassette, which is replaced by gRNA expression cassettes via single Golden Gate reaction (Fig. 1b, c). After the Golden Gate reaction, the recipient vector will contain all sgRNAs assembled together with corresponding *pU6* or *pU3* promoters and flanked by attL1 and attL2 sites for the final single Gateway LR reaction (Fig. 1d). It is important to mention that we refrained from using double, triple LR reaction schemes, because the single Gateway LR reaction in our hands always produced a higher number of colonies and has thus greatly increased chances of success under sub-optimal cloning conditions. Therefore, we decided to design all our final T-DNA vectors with attR1 and attR2 sites for robustness and high cloning efficiency. In the final LR reaction, all gRNA cassettes are recombined together in the destination T-DNA vector that contains the *Cas9* gene, promoter to drive *Cas9* expression and fluorescent seed selection cassette of choice (Fig. 1d; Table 1).

**Table 1:** T-DNA vectors for fluorescent seed selection

Destination vector	Addgene no.	Gateway adapters	Promoter::Cas	Plant selection	Bacterial selection
pRU051	167677	attR1–attR2	*PcUBi4-2*::*SpCas9*	FastRed	Spectinomycin
pRU052	167678	attR1–attR2	*PcUBi4-2*::*SpCas9*	FastGreen	Spectinomycin
pRU053	167679	attR1–attR2	*pEC1.2*::*SpCas9*	FastRed	Spectinomycin
pRU054	167680	attR1–attR2	*pEC1.2*::*SpCas9*	FastGreen	Spectinomycin
pRU321	167681	attR1–attR2	*PcUBi4-2*::*SaCas9*	FastRed	Spectinomycin
pRU320	167682	attR1–attR2	*PcUBi4-2*::*SaCas9*	FastGreen	Spectinomycin
pRU322	167683	attR1–attR2	*pEC1.2*::*SaCas9*	FastGreen	Spectinomycin
pRU055	167684	attR1–attR2	*pEC1.2*::*AsCas12a*^a^	FastRed	Spectinomycin
pRU205	167685	attR1–attR2	*PcUBi4-2*::*AsCas12a*	FastGreen	Spectinomycin
pRU319	167686	attR1–attR2	*pEC1.2*::*SpCas9*^a^	FastRed	Spectinomycin
pRU292	167687	attR1–attR2	*PcUBi4-2*::*zCas9i*	FastRed	Spectinomycin
pRU293	167688	attR1–attR2	*pUBi4-2*::*zCas9i*	FastGreen	Spectinomycin
pRU294	167689	attR1–attR2	*pEC1.2*::*zCas9i*	FastRed	Spectinomycin
pRU323	167690	attR1–attR2	*PcUBi4-2*::*zCas9i*	FastCyan	Spectinomycin
pRU324	167691	attR1–attR2	*pEC1.2*::*zCas9i*	FastCyan	Spectinomycin
pRU061	167692	attR1–attR2	*PcUBi4-2*::*AsCas12a*	FastRed	Spectinomycin
pRU206	167693	attR1–attR2	*pEC1.2*::*AsCas12a*	FastGreen	Spectinomycin
pRU295	167694	attR1–attR2	*pEC1.2*::*zCas9i*	FastGreen	Spectinomycin

T-DNA vectors for FastRed, FastGreen and FastCyan selection

^a^The vectors with all three elements replaceable using restriction sites

To this end, we constructed eight Golden Gate entry vectors harboring *pU6* or *pU3*-based cassettes (pRU41–pRU48) and six Golden Gate recipient vectors (pSF463, pSF464, pSF278, pSF279, pSF280 and pRU325) for testing assembly for up to eight gRNA cassettes into a single vector (Fig. 1a–c, Additional file 1: Tables S1, S2). We found assembly of all eight gRNAs cassettes via Golden Gate was easily achieved and the efficiency for the Golden Gate assembly into intermediate vectors was generally between 70 and 90% (between 7 and 9 colonies out of 10 had correct assembly of gRNAs). All the colonies generated after the final single Gateway LR reaction were positive (based on 10 independent single Gateway LR reactions).

The T-DNA vector module contains plasmids carrying *Cas9*, *zCas9i* or* Cas12a* variants that have been previously used in higher plants (Table 1; Additional file 2: Figure S1a). They are plant codon-optimized *SpCas9*, *SaCas9*, *zCas9i* and *AsCas12a* variants [13, 20, 30, 53]. The expression of the different Cas variants is driven either under *PcUBi4-2* or *pEC1.2* promoter [13, 31, 32]. Differently from *Cas9* and intronized *zCas9i*, *SaCas9* and *AsCas12a* have different requirements concerning the interaction between crRNA (CRISPR RNA) and tracrRNA (trans-activating crRNA) and previously developed entry vectors, such as pEn-Sa_Chimera and pYPQ133-STU-As (Addgene ID 138100) can be used for gRNA cloning [13, 54]. pEn-Sa_Chimera contains the *Arabidopsis *pU6-26 promoter, followed by the spacer for cloning the 20 nt sgRNA sequence using *BbsI* restriction sites and tracrRNA specific to *SaCas9*. This cassette is flanked by attL1-attL2 recombination sites for an easy single-step Gateway cloning into pRU320, pRU321 and pRU322 T-DNA vectors for fluorescent seed selection. For cloning into the vectors containing *AsCas12a*, pYPQ133-STU-As entry clone, developed by Zhang et al. can be used [54]. pYPQ133-STU-As contains *AsCas12a* crRNA scaffold flanked by the hammer head (HH) and hepatitis delta virus (HDV) ribozymes and a sgRNA cloning site between HH and HDV. Such cassette harboring different sgRNAs can be subcloned into pRU41-pRU48 vectors using *BbsI* restriction sites and used for cloning into intermediate vectors.

(pSF463, pSF464, pSF278, pSF279, pSF280 and pRU325) for Single-step Gateway reaction to be delivered into pRU055, pRU205, pRU061 and pRU295 T-DNA vectors for fluorescent seed selection.

As illustrated in the Fig. 1, our assembly of a multiplex CRISPR/Cas9 T-DNA vector takes three steps and requires very basic molecular biology techniques. Importantly, PCR is not used for any cloning step, which reduces the likelihood that mutations will occur within the CRISPR/Cas9 components and obviates the need for control sequencing. Having established the system, we next tested our vector systems for genome editing. The following work mainly focuses on testing vectors expressing *Cas9* and z*Cas9i* under *pEC1.2* or *PcUBi4-2* promoters.

### The game of colors—a cocktail of FastRed and FastGreen vectors for a faster screening of editing events

Into each T-DNA vector carrying the *Cas9* cassette we have introduced *pOLE1*::*OLE1* fusion tagged either with GFP (FastGreen), tagRFP (FastRed) or mTurquoise2 (FastCyan) to be able to screen seeds with red, green or cyan fluorescence simply by using a fluorescent stereomicroscope (Fig. 1d; Table 1; Additional file 1: Table S1A). As described previously, FAST (fluorescence-accumulating seed technology) is based on the expression of OLE1 (OLEOSIN1) translational fusions, under the control of its native pOLE1 promoter, thus accumulating fluorescence on the oil body membranes in the developing seeds of *Arabidopsis *[56]. *Arabidopsis *seeds accumulate a large quantity of oil bodies, which are surrounded by phospholipid membranes with embedded proteins within the cell [56]. Oleosins are abundant structural proteins embedded in oil body membranes, have an important function in regulating the size of oil bodies and confer freezing tolerance upon seeds [55]. Among all oleosins, OLE1 is the most abundant in *Arabidopsis *seeds [57, 58].

The aim of using fluorescent seed selection was to reduce the length of time for: (1) identifying the transgenic lines in T1 carrying the desired events; (2) obtaining homozygous, *CAS9*-free mutants in T2 generation.

**Fig. 2:**
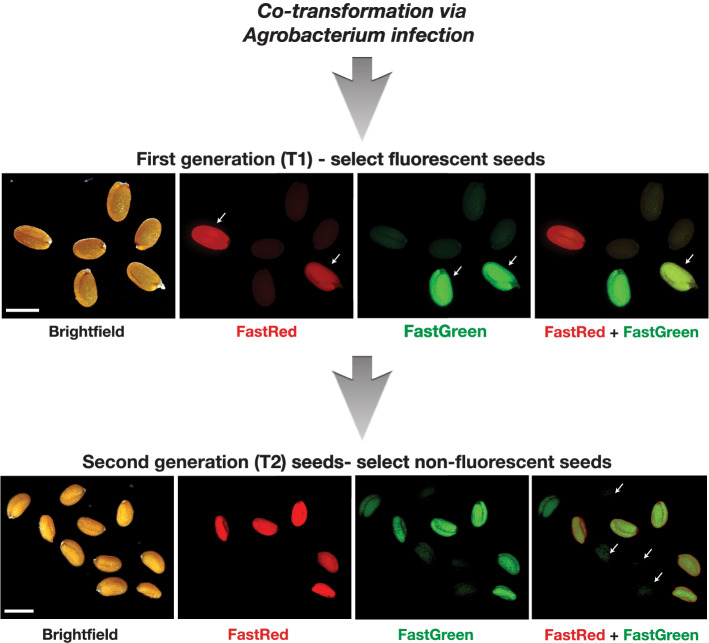
Fluorescent seed selection in T1 and T2 generations after *Agrobacterium*-mediated transformation. Green, red or yellow (two color) fluorescent seeds are selected in T1 generation, genotyped for editing events and the non-fluorescent, *Cas9*-free, seeds of T2 progenies are screened for stably inherited mutations. Scale bars  = 100 μm

In addition, we decided to test whether the T-DNA constructs, carrying FastRed or FastGreen, can be combined together via simple *Agrobacterium*-based co-transformation. Immediately before dipping *Arabidopsis *flowers, we mixed the two *Agrobacterium* cultures in equal proportions together into one cocktail. In T1 generation, we were able to screen either for red or green fluorescent seeds, or both colors together (rates of 3–4% single color seeds and 0.5–1% of two-color seeds, based on four independent co-dipping experiments, 16 plants co-dipped in each experiment). Dependent on the filter set available in the users’ fluorescent stereomicroscopes, the seeds can be screened for both colors either by switching the filters in-between or by using the long pass filters for green fluorescence to select the yellow color seeds. In T2 generation, we noticed that around 70% of independent two-color seed lines contained mostly yellow seeds (between 33 and 95% of total number of seeds) suggesting that, although coming from two separate vectors and independent bacteria, FastRed and FastGreen cassettes containing T-DNAs often enter into a common locus and co-segregate together (Fig. 2; Additional file 2: Figure S1b). Screening of non-fluorescent seeds in T2 (counter-selection) allowed us to obtain *Cas9*-free homozygous mutants. We used *cuc1cuc2* double mutants as examples to demonstrate the application of our FastRed and FastGreen co-transformation system.

### Simultaneous targeting of different *Arabidopsis loci* using FastRed and FastGreen strategy

**Fig. 3:**
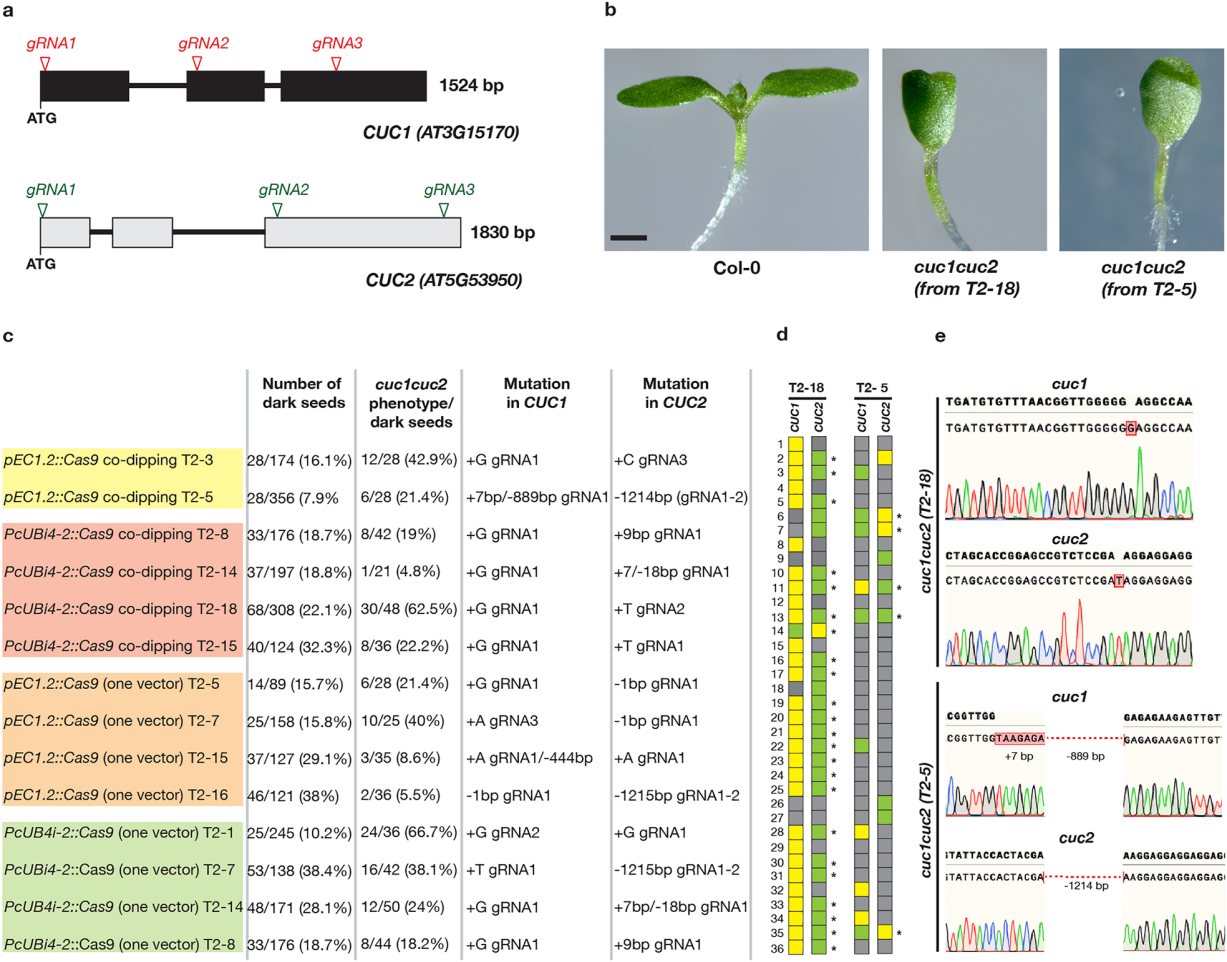
Example of the application of co-dipping strategy. **a** Graphical representation of *CUC1* and *CUC2* genes and positions of gRNAs. **b** *cuc1cuc2* knockout cotyledon phenotype in the isolated *pEC1.2*::*Cas9* (T2-5) and *PcUBi4-2*::*Cas9* (T2-18) lines. **c** Segregation analysis of the selected T2 lines. Counting the non-fluorescent seeds and evaluating *cuc1cuc2* phenotype among the non-fluorescent seeds were performed independently. **d** Segregation analysis in 36 wild-type looking individuals derived from non-fluorescent seeds from the selected *pEC1.2*::*Cas9* (T2-5) and *PcUBi4-2*::*Cas9* (T2-18) lines. Yellow color indicates homozygous, green indicates heterozygous and grey color indicates wild-type individuals. The asterisks indicate the individuals which are homozygous for one of the two genes and heterozygous for the other one. **e** The chromatograms showing the type of mutations in *pEC1.2*::*Cas9* (T2-5) and *PcUBi4-2*::*Cas9* (T2-18) lines as identified by sequencing. Scale bar in **b**  =  1 mm

First, we tested our system for simultaneously creating targeted deletions in *Arabidopsis CUC1* and *CUC2* loci. The *CUP-SHAPED COTYLEDON* genes *CUC1* and *CUC2* encode a pair of NAC transcription factors required for shoot meristem initiation. They are functionally redundant and the seedlings of each single mutant show little morphological phenotype while the double mutant completely lacks a shoot meristem and produce completely fused cotyledons [59–62]. To increase the chance of obtaining different mutant alleles and large deletions, we picked three sgRNAs for each, *CUC1* and *CUC2*, located in different exons and distributed along the coding sequence (Fig. 3a). The three sgRNAs for each gene were expressed either under the *pU6* or *pU3* promoter as indicated in the cloning setup (Fig. 1a).

The T-DNA expression vector for *CUC1* contained FastRed and the vector for *CUC2—*FastGreen selection marker. To compare the efficiencies, we used constructs containing either *pEC1.2* or *PcUBi4-2* promoter driving *Cas9* expression. Both, FastRed and FastGreen constructs, were co-dipped together as an *Agrobacterium* cocktail into wild-type Col-0 plants to generate seeds with single or both colors. To verify the stability of these vectors, we sequenced and verified all the elements before and after transformation into *Agrobacterium*. In the first generation after transformation (T1), we selected the fluorescent seeds containing both colors using fluorescent stereomicroscope, germinated them on plates and genotyped. We identified heterozygous (3/19; 15.8% for *pEC1.2* and 6/22; 22.7% for *PcUBi4-2* construct) and biallelic (3/19; 15.8% efficiency for *pEC1.2* and 3/22; 11.1% for *PcUBi4-2* constructs) double mutants of *cuc1cuc2* (Fig. 3b). We also identified 1/19 (5.3%) and 3/22 (13.6%) chimeric plants in *pEC1.2* and *PcUBi4-2* experiments, accordingly. Although the overall number of events was higher in the co-transformation experiments where the *PcUBi4-2* was used to drive *Cas9*, we noticed that more biallelic mutants were found in T1 when *pEC1.2* promoter was used. This is consistent with previous studies where *pEC1.2* was shown to produce biallelic and heritable events already in T1.

As expected, the *cuc1cuc2* double mutants exhibited a severe shoot phenotype with fused cotyledons (Fig. 3b). In our hands, the *cuc1cuc2* mutants were not viable and therefore we had to maintain heterozygous T1 lines. To check if the detected mutations in both genes were stably transmitted from T1 to T2 generation, we took two T1 lines from *pEC1.2*::*Cas9* and four lines from *PcUBi4-2*::*Cas9* co-dipping experiment which were heterozygous for one mutation and homozygous for the other, left them to self-pollinate, selected the non-fluorescent seeds and checked the segregation and type of mutations in the following T2 generation. The resulting T2 lines segregated and we were able to detect a high rate of seedlings showing double mutant phenotype.

We sorted 36 non-fluorescent seeds of two T2 lines, one derived from *pEC1.2*::*Cas9* (T2-5) and the other one from *PcUBi4-2*::*Cas9* (T2-18) co-dipping experiments, for more detailed segregation analysis. In case of *pEC1.2*::*Cas9* (T2-5) line, we detected 5/36 individuals (13.9%) with a heterozygous mutation for one of the *CUC* genes and homozygous for the other gene. In case of *PcUBi4-2*::*Cas9* (T2-18) line, we found 23/36 individuals (63.9%) heterozygous for one of the *CUC* genes and homozygous for the other gene (Fig. 3d, e). We further verified the absence of *Cas9* by PCR using *Cas9*-specific primers in these lines. Thus, following all these results, our multiplex CRISPR/Cas9 system allows to use at least three sgRNAs/gene and the combination of two constructs to target two genomic loci simultaneously. Such strategy also provides flexibility in the screening strategy where generation of different combinations of mutants is required (e.g., generation to two pairs of double mutant, in addition to a quadruple mutant).

To test if we can efficiently target a higher number of sgRNAs using the same construct, we cloned three sgRNAs for *CUC1* and two sgRNAs for *CUC2* into the same vector carrying *Cas9* under *pEC1.2* promoter. The resulting vector contained five gRNA cassettes in total. In T1, we could obtain biallelic *cuc1cuc2* (16%; 4/25 for *pEC1.2*::*Cas9* and 14.3%; 4/28 for *PcUBi4-2*::*Cas9*), heterozygous (5/25; 25% for *pEC1.2*::*Cas9* and 7/28; 25% for *PcUBi4-2*::*Cas9*) with similar ratio compared to the co-transformation strategy. This suggest that stacking up at least five gRNAs into the same vector does not affect their efficiency. In T2 generation, we were able to identify a number of segregating lines containing *Cas9*-free and carrying different types of mutations (Fig. 3c). Using the co-dipping technique and stacking up to five gRNAs into one vector we obtained mostly point mutations (about 85% for *CUC1* and 50% for *CUC2*, based on 14 independent T2 lines), occasionally large deletions (about 5% for *CUC1* and 21% for *CUC2*, based on 14 independent T2 lines) and small deletions or insertions (about 29% for *CUC2* and none detected for *CUC1*, based on 14 independent T2 lines).

Given that the vectors we generated have maximum capacity of eight gRNAs, the number can be doubled to sixteen using co-transformation technique. This provides a powerful tool for generation of high order *Arabidopsis *mutants. The system described here has been successfully applied to generate several important high order *Arabidopsis *mutants: quintuple *gelp22/gelp38/gelp49/gelp51/gelp96* (*gelp*^*quint*^) mutant lacking the core root endodermis suberin polymerization machinery, nonuple endodermis-specific laccase *lac1;3;5;7;8;9;12;13;16 (*9 × *lac)* mutant and a quadruple *myb41-myb53-myb92-myb93 (quad-myb)* with mutations in four transcription factors essential for promoting endodermal suberin formation [63–65]. In case of *gelp*^*quint*^, a co-dipping strategy was employed using two different FastRed and Fastgreen vectors which were harboring in total 10 sgRNAs to target five endodermis-specific GELP genes (two sgRNAs/gene). Using this strategy, we were able to efficiently obtain different order *gelp* mutants, including two different *gelp*^*quint*^ alleles by screening the non-fluorescent seeds from twelve different T2 lines (96 individuals in total). Although we could not detect large deletions in *gelp*^*quint*^ screen using two sgRNAs/gene, we were able to identify small events, such as single nucleotide insertions, deletions or few nucleotide deletions in either of the two sgRNAs or both [65]. This suggests that choosing at least two sgRNAs to target one gene increases the chances of successful gene editing, as well as obtaining different multiple mutant alleles.

### Flexible CRISPR/Cas T-DNA vectors for further modification and improvement

**Fig. 4:**
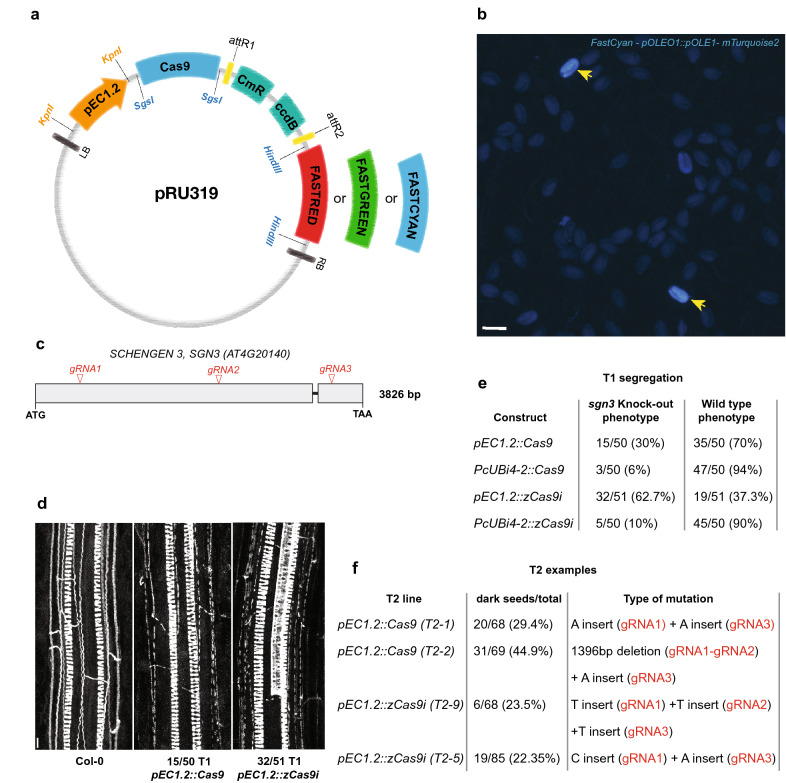
CRISPR/Cas T-DNA vectors for further modification and improvement. **a** Schematic representation of T-DNA vector (pRU319) containing *KpnI*, *SgsI* and *HindIII* restrictions sites flanking the promoter, Cas gene and selection cassette, accordingly. **b** FastCyan seed selection based on seed-specific *pOLE*::*OLE1-mTurquoise2* expression. Yellow arrows indicate the positive fluorescent seeds. **c** Schematics of *SGN3* gene showing the positions of the chosen sgRNAs. **d** Basic Fuchsin (gray) staining of lignin-based Casparian strips in the isolated T1 generation biallelic *sgn3* mutants generated using *pEC1.2*::*Cas9* and *pEC1.2*::*zCas9i* constructs. **e** Segregation analysis in T1 generation in *pEC1.2*::*Cas9*, *pEC1.2*::*zCas9i*, *PcUBi4-2*::*Cas9* and *PcUBi4-2*::*zCas9i* lines. **f** Segregation analysis of T2 generation in *pEC1.2*::*Cas9* and *pEC1.2*::*zCas9i* lines. Scale bars  = 100 μm (**B**) and in 25 μm (**D**)

Currently, new Cas proteins and their modifications are being discovered and reported at a high rate [7, 13, 15–17]. Some of them are more efficient than others, with different binding properties and different PAM sites. Therefore, we decided to modify some of our vectors in order to enable a fast and easy swapping of the promoter to drive Cas, Cas itself and the plant selection cassette. The vectors pRU055 and pRU319 have *KpnI* restriction sites flanking the promoter *pEC1.2*, as well as *SgsI* or *Asc*I sites flanking the *Cas9* sequence and *HindIII* restriction sites flanking the fluorescent seed selection cassette (Fig. 4a). All these restriction sites allow for a fast and efficient replacement of all three elements in the future. Vectors pRU051, pRU052, pRU292, pRU293, pRU294 and pRU295 allow an easy replacement of *Cas9* and selection cassette. Our vectors could be easily modified for generating tissue-specific CRISPR by replacing *PcUBi4-2* or *pEC1.2* with a tissue-specific promoter of interest, for example.

To demonstrate the advantage of our modular cloning system, we decided to replace the *Cas9* in the destination vector *pEC1.2*::*SpCas9* (*pRU319*) with a recently published optimized version of *Cas9, zCas9i*, containing 13 introns and two NLS signals flanking the z*Cas9*. *zCas9i* has been reported to dramatically improve the editing efficiency already in the primary T1 transformants. According to Grützner et al., none of the primary transformants obtained with a *zCas9* lacking introns driven under *pRPS5a* promoter displayed a knockout mutant phenotype, whereas between 70 and 100% of the transformants generated with the *zCas9i* displayed mutant phenotypes [20]. Since in our hands, non-modified *Cas9* is much more efficient, we wondered what degree of improvement in efficiency of *zCas9i* we would obtain in our system with a better-working, unmodified *Cas9*.

In addition to z*Cas9i*, we also replaced the FastRed cassette with FastGreen and a newly generated FastCyan to produce a set of destination vectors containing *zCas9i* driven either under *pEC1.2* or *PcUBi4-2* and combined with FastRed, FastGreen or FastCyan fluorescent seed selection markers (Fig. 4b; Table 1; Additional file 2: Figure S1). We introduced the FastCyan-based fluorescent seed selection cassette to be able to sort the seeds not only in the red and green spectrum, but also in the cerulean spectrum. To test the efficiency of these newly generated plasmids, we chose *SGN3 (SCHENGEN 3)* as a target gene. *SGN3* encodes a receptor-like kinase with an important function in maintaining the integrity of Casparian strips, lignin-based barrier in the root endodermis. In the absence of SGN3, discontinuous patches of lignin can be easily observed using Basic Fuchsin staining [66–68]. For comparison, we introduced three sgRNAs to target SGN3 into the vectors containing z*Cas9i* and *Cas9* lacking the introns (Fig. 4c). In both cases, *Cas9* or z*Cas9i* were driven either under *PcUBi4-2* or *pEC1.2* promoters. In T1 generation, we assessed the number of *sgn3* knockout phenotype appearing in different lines. We observed that the highest number of knockout phenotypes (32/51, 62.7%) were displayed by the T1 line generated using intron-containing *zCas9i* driven under *pEC1.2* promoter. In comparison, the same promoter driving regular, intron-less *Cas9*, generated 30% (15/50) knockout phenotype displaying individuals (Fig. 4d-e). Although *zCas9i* driven under ubiquitous *PcUBi4-2* promoter generated significantly lower number of knockouts in T1 generation (5/50, 10%), it was still higher compared to the same promoter driving regular *Cas9* (3/50, 6%) (Fig. 4e). To check the inheritance of the knockout phenotypes, we generated T2 lines and checked two lines from each construct, *pEC1.2*::*Cas9* and *pEC1.2*::*zCas9*i. In all four lines we could confirm homozygous *sgn3* phenotype by selecting non-fluorescent, *Cas9*-free, seeds (Fig. 4f). These results show that *zCas9i* indeed represents a significant improvement over intron-less variants, even when compared in a system in which intron-less *Cas9* shows a reasonable efficiency. Our data indicates that the *pEC1*::*zCas9i* construct reported here is a highly efficient vector for the generation of biallelic mutants already in T1 generation.

## Conclusions

In this study, we developed and tested a two-color based multiplex CRISPR/Cas9 toolbox that consists of Golden Gate- and Gateway-compatible vectors, all of which are available at Addgene (https://www.addgene.org). The vectors allow to assemble up to eight (sixteen in case of co-dipping) gRNAs. The T-DNA vectors we developed contain either FastRed, FastGreen or FastCyan cassette which: (1) simplifies the cloning strategy by avoiding the generation of very large constructs; (2) simplifies the screening method in the first generation (T1) where only fluorescent seeds are selected, as well as in second generation (T2) where *Cas9* presence is counter-selected by picking non-fluorescent seeds and which are then checked for the desired editing events; (3) provides ability to easily screen for different, higher order mutants in the same generation. To demonstrate multiplexing, we cloned three independent gRNAs in each vector simultaneously, co-transformed them together into Arabidopsis, an approach that has not yet been explored. The T-DNA vectors contain either *pEC1.2* or *PcUBi4-2* promoter to drive *Cas9*, intron-containing z*Cas9i, SaCas9* or *AsCas12a* and fluorescent seed selection cassette of choice—FastRed, FastGreen or FastCyan. Moreover, most of the vectors we generated can be easily modified to be able to introduce any promoter, Cas gene or selection cassette of interest. Although we could occasionally obtain large deletions by using three sgRNAs to target the same gene, most of the detected mutations were either single nucleotide insertions or deletions. However, we demonstrated here and with an example of generating the quintuple *gelp* mutant that, by increasing the number of sgRNAs, we increase the chance of choosing the more efficient sgRNAs for gene editing and obtaining different multiple mutant alleles. Further research on how the distance between sgRNAs affects the chance of obtaining higher number of large deletions, is required.

In summary, we believe that the toolbox presented here will be very useful in plant research and plant synthetic biology, due to its streamlined, easy-to-use and efficient cloning and selection system. Moreover, its modularity and flexibility will allow researcher to easily build-on and improve the system in the future.

## Materials and methods

### Plant growth and transformation

In all experiments *Arabidopsis thaliana* Columbia (Col-0) ecotype was used. The seedlings were germinated on solid half-strength Murashige and Skoog (MS) medium without addition of sucrose. *CUC1 *(*At3g15170*), *CUC2 *(*At5g53950*) and *SGN3 *(*AT4G20140*) genes were chosen as targets for CRISPR/Cas9 targeting. The seeds of T1 and T2 generations were surface sterilized, sown on plates, incubated for 2 days at 4 °C for stratification, and grown vertically in growth chambers at 22 °C, under continuous light (100 μE). The phenotypic analyses were performed on 6-day-old seedlings. For *Agrobacterium*-mediated transformation, siliques of flowering plants were removed and a solution of resuspended *Agrobacterium* cells carrying corresponding CRISPR constructs with sucrose and SILWETT (5% of sucrose and 0.06% Silwet L-77) was directly applied to flower buds by pipetting. In case of co-transformation, FastRed and FastGreen vectors were transformed separately into *Agrobacterium* and grown overnight in 5 ml cultures at 28 °C. The cultures were centrifuged for 10 min at 4000 rpm, the pellets resuspended in sucrose and Silwet L-77 solution. The resuspended FastRed and FastGreen pellets were mixed in equal amount to make a cocktail for transforming both constructs at same time.

### Generation of CRISPR/Cas9 vectors

The primers used to generate all vectors are indicated in Additional file 1: Table S1. pChimera (Addgene ID 61432) [30] was used as a template to generate pRU41, pRU43, pRU45 and pRU47 vectors (Additional file 1: Table S1). The pRU42, pRU44, pRU46 and pRU48 were generated by replacing the *pU6* promoter with *pU3*. The corresponding *BsaI* sites were introduced to generate compatible overhangs in all the entry clones as shown in Fig. 1. The intermediate vectors pSF463, pSF278, pSF464, pSF279, pSF280, pSF325 were generated by introducing the corresponding *Bsa*I sites into pDONR221 containing ccdB cassette and flanking attL1 and attL2 recombination sites ready for single fragment Gateway cloning (Additional file 1: Table S2). The final T-DNA vectors were generated using pDe-Cas9 as template (Addgene ID 61433) [30]. The FastRed, FastGreen and FastCyan selection cassettes were amplified from pFRm43GW (Addgene ID 133748), pFG7m34GW (Addgene ID 133747) [69] and *UBQ*::*NLS-mTurquoise2* [70] vectors and introduced into pDe-Cas9 vector in place of PPT selection using *HindIII* (FastDigest, Thermo Fisher Scientific, Catalog Number FD0504) restriction sites. Different *Cas9* and* Cas12a* variants, as well as *zCas9i* were amplified from vectors pDe-SaCas9 [13], pYPQ220 (Addgene ID 86208) [53] and pAGM47523 (Addgene ID 153221) [20] and introduced by replacing *Cas9* using *SgsI* (FastDigest, Thermo Fisher Scientific, Catalog Number FD1894) restriction sites in pDe-Cas9 vector. *pEC1.2* was amplified from pHEE401E (Addgene ID 71287) [31] vector and introduced into different vectors by replacing the *PcUBi4-2* promoter vector using *KpnI* (FastDigest, Thermo Fisher Scientific, Catalog Number FD0524) restriction sites. All vectors generated in this study are shown in Table 1; Additional file 1: Tables S1, S2 and are deposited at Addgene plasmid repository (https://www.addgene.org). The primers used for generating modification of the vectors are shown in Additional file 1: Table S3. The primers for introducing the required gRNAs into the entry vectors are indicated in Additional file 1: Table S4 and the detailed procedure of generating T-DNA vectors carrying the gRNAs is described in Additional file 3: Materials and methods. Benchling (https://www.benchling.com) platform was used to find the optimal sgRNAs. The sites of the chosen sgRNAs are indicated in the Fig. 3 (for *CUC1* and *CUC2* genes) and Fig. 4 (for *SGN3* gene). The sgRNA target sites were always located in exons with the first sgRNA in close proximity to translation start site.

### Screening of CRISPR mutants

Fluorescent seeds of T1 plants carrying FastGreen, FastRed and FastCyan cassettes, as well as non-fluorescent seeds of T2 lines were screened using Leica MZ16FA Fluorescence Stereomicroscope. The filters used for different colours are as follows: DSR (LEICA 10447227) for FastRed; GFP3 (LEICA 10447217) for FastGreen; CFP (LEICA 10447409) for FastCyan; GFP2 (LEICA 10447221) for yellow seeds carrying both FastRed and FastGreen constructs.

Genomic DNA of transgenic CRISPR T1 and non-transgenic T2 plants was extracted using CTAB method [71]. The leaves and flowers were used for DNA extraction. The plant material was crushed using pipette tips directly in 100 μl CTAB buffer and incubated for 40 min at 65 °C. 100 μl of chloroform/isoamyl alcohol (16/1 ratio) was added, mixed by inverting and centrifuged for 5 min at max speed. The upper phase was collected, mixed with 50 μl of isopropanol and incubated overnight. Next day, the samples were centrifuged for 10 min at maximum speed. The liquid was discarded and, after drying, the pellet was resuspended in 50 μl of water. Primers used for genotyping and sequencing are indicated in Additional file 1: Tables S5; S6.

### Lignin staining and confocal microscopy

ClearSee‐adapted Basic Fuchsin staining for lignin was performed as described earlier [72]. Confocal pictures of Basic Fuchsin stained *sgn3* mutant roots were obtained using Zeiss LSM 880 confocal microscope. The excitation and emission spectra for Basic Fuchsin are 561 nm and 570–650 nm accordingly.

## Supplementary Information

**Additional file 1: Table S1.** 1st level intermediate vectors containing the corresponding BsaI for GoldenGate -based assembly. **Table S2**. 2nd level intermediate vectors. Please note that these vectors contain ccdB cassette and ccdB survival competent cells combined with Kanamycin and Chloramphenicol have to be used to propagate the empty vectors. **Table S3. **Primers for generation and modification of CRISPR vectors. **Table S4. **List of primers designed for gRNAs used in this study (overhangs for oligo annealing without leaving a scar after pU6 or pU3 promoters are highlighted in red). **Table S5. **Primers used for PCR-based genotyping. **Table S6. **List of the primers used for *Cas9* amplification.

**Additional file 2: Figure S1. **T-DNA vectors developed in this study. **a** Schematics showing the collection of final T-DNA vectors developed in this study. **b** Example of dominant presence of double-color fluorescent seeds in T2 generation. Scale bar = 100 μm.

**Additional file 3: Materials and methods.** CRISPR/Cas9 cloning protocol

## Data Availability

Vectors and seeds generated in this study can be requested from the corresponding authors. All the vectors are also available via Addgene (https://www.addgene.org). The corresponding names and numbers are indicated in Table 1, Additional file 1: Tables S1, S2.

## References

[1] Weinthal D, Tovkach A, Zeevi V, Tzfira T (2010). Genome editing in plant cells by zinc finger nucleases. Trends Plant Sci.

[2] Curtin SJ, Voytas DF, Stupar RM (2012). Genome engineering of crops with designer nucleases. Plant Genome.

[3] Malzahn A, Lowder L, Qi Y (2017). Plant genome editing with TALEN and CRISPR. Cell Biosci.

[4] Atkins PA, Voytas DF (2020). Overcoming bottlenecks in plant gene editing. Curr Opin Plant Biol.

[5] Schindele A, Dorn A, Puchta H (2020). CRISPR/Cas brings plant biology and breeding into the fast lane. Curr Opin Biotechnol.

[6] Zhang D, Hussain A, Manghwar H, Xie K, Xie S, Zhao S (2020). Genome editing with the CRISPR-Cas system: an art, ethics and global regulatory perspective. Plant Biotechnol J.

[7] Huang T-K, Puchta H (2021). Novel CRISPR/Cas applications in plants: from prime editing to chromosome engineering. Transgenic Res.

[8] Liu L, Fan X-D (2014). CRISPR–Cas system: a powerful tool for genome engineering. Plant Mol Biol.

[9] Ma X, Zhu Q, Chen Y, Liu Y-G (2016). CRISPR/Cas9 platforms for genome editing in plants: developments and applications. Mol Plant.

[10] Manghwar H, Lindsey K, Zhang X, Jin S (2019). CRISPR/Cas system: recent advances and future prospects for genome editing. Trends Plant Sci.

[11] Gao C (2021). Genome engineering for crop improvement and future agriculture. Cell.

[12] Sukegawa S, Saika H, Toki S (2021). Plant genome editing: ever more precise and wide reaching. Plant J.

[13] Steinert J, Schiml S, Fauser F, Puchta H (2015). Highly efficient heritable plant genome engineering using *Cas9* orthologues from *Streptococcus thermophilus* and *Staphylococcus aureus*. Plant J.

[14] Steinert J, Schmidt C, Puchta H (2017). Use of the *Cas9* orthologs from *Streptococcus**thermophilus* and *Staphylococcus**aureus* for non-homologous end-joining mediated site-specific mutagenesis in *Arabidopsis thaliana*. Methods Mol Biol.

[15] Schindele P, Puchta H (2019). Engineering CRISPR/*Lb Cas12a* for highly efficient, temperature-tolerant plant gene editing. Plant Biotechnol J.

[16] Wolter F, Puchta H (2019). In planta gene targeting can be enhanced by the use of CRISPR/Cas12a. Plant J.

[17] Merker L, Schindele P, Huang T, Wolter F, Puchta H (2020). Enhancing in planta gene targeting efficiencies in *Arabidopsis* using temperature-tolerant CRISPR/ *Lb Cas12a*. Plant Biotechnol J.

[18] Doudna JA, Charpentier E. The new frontier of genome engineering with CRISPR-Cas9. Science. American Association for the Advancement of Science. 2014. https://science.sciencemag.org/content/346/6213/1258096. Accessed 7 Mar 2020. 10.1126/science.1258096 25430774

[19] Capdeville N, Merker L, Schindele P, Puchta H (2021). Sophisticated CRISPR/Cas tools for fine-tuning plant performance. J Plant Physiol.

[20] Grützner R, Martin P, Horn C, Mortensen S, Cram EJ, Lee-Parsons CWT (2021). High-efficiency genome editing in plants mediated by a *Cas9* gene containing multiple introns. Plant Commun.

[21] Clough SJ, Bent AF (1998). Floral dip: a simplified method for *Agrobacterium*-mediated transformation of *Arabidopsis**thaliana*: floral dip transformation of *Arabidopsis*. Plant J.

[22] Newell CA (2000). Plant transformation technology: developments and applications. MB.

[23] Gelvin SB (2003). *Agrobacterium*-mediated plant transformation: the biology behind the “gene-jockeying” tool. Microbiol Mol Biol Rev.

[24] Zhang X, Henriques R, Lin S-S, Niu Q-W, Chua N-H (2006). *Agrobacterium*-mediated transformation of *Arabidopsis**thaliana* using the floral dip method. Nat Protoc.

[25] Hwang H-H, Yu M, Lai E-M (2017). *Agrobacterium*-mediated plant transformation: biology and applications. Arabidopsis Book.

[26] Stuitje AR, Verbree EC, Van Der Linden KH, Mietkiewska EM, Nap J-P, Kneppers TJA (2003). Seed-expressed fluorescent proteins as versatile tools for easy (co)transformation and high-throughput functional genomics in *Arabidopsis*: seed-expressed fluorescent proteins in *Arabidopsis*. Plant Biotechnol J.

[27] Schmidt R, Willmitzer L, Lindsey K (1991). *Arabidopsis* regeneration and transformation (leaf & cotyledon explant system). Plant tissue culture manual.

[28] Clarke MC, Wei W, Lindsey K (1992). High-frequency transformation of* Arabidopsis**thaliana* by* Agrobacterium**tumefaciens*. Plant Mol Biol Rep.

[29] Bent AF (2000). *Arabidopsis* in planta transformation uses, mechanisms, and prospects for transformation of other species. Plant Physiol.

[30] Fauser F, Schiml S, Puchta H (2014). Both CRISPR/Cas-based nucleases and nickases can be used efficiently for genome engineering in *Arabidopsis thaliana*. Plant J.

[31] Wang Z-P, Xing H-L, Dong L, Zhang H-Y, Han C-Y, Wang X-C (2015). Egg cell-specific promoter-controlled CRISPR/Cas9 efficiently generates homozygous mutants for multiple target genes in *Arabidopsis* in a single generation. Genome Biol.

[32] Wolter F, Klemm J, Puchta H (2018). Efficient in planta gene targeting in *Arabidopsis* using egg cell-specific expression of the *Cas9* nuclease of *Staphylococcus aureus*. Plant J.

[33] Yan L, Wei S, Wu Y, Hu R, Li H, Yang W (2015). High-efficiency genome editing in *Arabidopsis* using YAO promoter-driven CRISPR/Cas9 system. Mol Plant.

[34] Mao Y, Zhang Z, Feng Z, Wei P, Zhang H, Botella JR (2016). Development of germ-line-specific CRISPR-Cas9 systems to improve the production of heritable gene modifications in *Arabidopsis*. Plant Biotechnol J.

[35] Bandyopadhyay A, Kancharla N, Javalkote VS, Dasgupta S, Brutnell TP (2020). CRISPR-Cas12a (Cpf1): a versatile tool in the plant genome editing tool box for agricultural advancement. Front Plant Sci.

[36] Schiml S, Fauser F, Puchta H (2014). The CRISPR/Cas system can be used as nuclease for in planta gene targeting and as paired nickases for directed mutagenesis in *Arabidopsis* resulting in heritable progeny. Plant J.

[37] Schmidt C, Pacher M, Puchta H (2019). Efficient induction of heritable inversions in plant genomes using the CRISPR /Cas system. Plant J.

[38] Cong L, Ran FA, Cox D, Lin S, Barretto R, Habib N (2013). Multiplex genome engineering using CRISPR/Cas systems. Science.

[39] Ran FA, Hsu PD, Wright J, Agarwala V, Scott DA, Zhang F (2013). Genome engineering using the CRISPR-Cas9 system. Nat Protoc.

[40] Engler C, Kandzia R, Marillonnet S, El-Shemy HA (2008). A one pot, one step, precision cloning method with high throughput capability. PLoS ONE.

[41] Earley KW, Haag JR, Pontes O, Opper K, Juehne T, Song K (2006). Gateway-compatible vectors for plant functional genomics and proteomics. Plant J.

[42] Curtis MD, Grossniklaus U (2003). A gateway cloning vector set for high-throughput functional analysis of genes in planta. Plant Physiol.

[43] Karimi M, De Meyer B, Hilson P (2005). Modular cloning in plant cells. Trends Plant Sci.

[44] Karimi M, Depicker A, Hilson P (2007). Recombinational cloning with plant gateway vectors. Plant Physiol.

[45] Aboulela M, Tanaka Y, Nishimura K, Mano S, Nishimura M, Ishiguro S, Ng C (2017). Development of an R4 dual-site (R4DS) gateway cloning system enabling the efficient simultaneous cloning of two desired sets of promoters and open reading frames in a binary vector for plant research. PLoS ONE.

[46] Luo Y, Qiu Y, Na R, Meerja F, Lu QS, Yang C (2018). A Golden Gate and Gateway double-compatible vector system for high throughput functional analysis of genes. Plant Science.

[47] Karimi M, Jacobs TB (2021). GoldenGateway: a DNA assembly method for plant biotechnology. Trends Plant Sci.

[48] Lowder LG, Zhang D, Baltes NJ, Paul JW, Tang X, Zheng X (2015). A CRISPR/Cas9 toolbox for multiplexed plant genome editing and transcriptional regulation. Plant Physiol.

[49] Kim H, Kim S-T, Ryu J, Choi MK, Kweon J, Kang B-C (2016). A simple, flexible and high-throughput cloning system for plant genome editing via CRISPR-Cas system: single CRISPR-Cas binary vector for plant genome editing. J Integr Plant Biol.

[50] Lowder L, Malzahn A, Qi Y, Shan L, He P (2017). Rapid construction of multiplexed CRISPR-Cas9 systems for plant genome editing. Plant pattern recognition receptors.

[51] Denbow CJ, Lapins S, Dietz N, Scherer R, Nimchuk ZL, Okumoto S (2017). Gateway-compatible CRISPR-Cas9 vectors and a rapid detection by high-resolution melting curve analysis. Front Plant Sci.

[52] Gasparis S, Kała M, Przyborowski M, Łyżnik LA, Orczyk W, Nadolska-Orczyk A (2018). A simple and efficient CRISPR/Cas9 platform for induction of single and multiple, heritable mutations in barley (*Hordeum**vulgare* L.). Plant Methods.

[53] Tang X, Lowder LG, Zhang T, Malzahn AA, Zheng X, Voytas DF (2017). A CRISPR–Cpf1 system for efficient genome editing and transcriptional repression in plants. Nature Plants.

[54] Zhang Y, Ren Q, Tang X, Liu S, Malzahn AA, Zhou J (2021). Expanding the scope of plant genome engineering with* Cas12a* orthologs and highly multiplexable editing systems. Nat Commun.

[55] Siloto RMP, Findlay K, Lopez-Villalobos A, Yeung EC, Nykiforuk CL, Moloney MM (2006). The accumulation of oleosins determines the size of seed oilbodies in *Arabidopsis*. Plant Cell.

[56] Huang AHC (1992). Oil bodies and oleosins in seeds. Annu Rev Plant Physiol Plant Mol Biol.

[57] Shimada TL, Shimada T, Takahashi H, Fukao Y, Hara-Nishimura I (2008). A novel role for oleosins in freezing tolerance of oilseeds in *Arabidopsis thaliana*. Plant J.

[58] Shimada TL, Shimada T, Hara-Nishimura I (2010). A rapid and non-destructive screenable marker, FAST, for identifying transformed seeds of *Arabidopsis thaliana*. Plant J.

[59] Aida M, Ishida T, Tasaka M (1999). Shoot apical meristem and cotyledon formation during *Arabidopsis**embryogenesis*: interaction among the CUP-SHAPED COTYLEDON and SHOOT MERISTEMLESS genes. Development.

[60] Aida M, Vernoux T, Furutani M, Traas J, Tasaka M (2002). Roles of PIN-FORMED1 and MONOPTEROS in pattern formation of the apical region of the *Arabidopsis* embryo. Development.

[61] Hibara K, Takada S, Tasaka M (2003). *CUC1* gene activates the expression of SAM-related genes to induce adventitious shoot formation: role of *CUC1* in adventitious SAM formation. Plant J.

[62] Hibara K, Karim MR, Takada S, Taoka K, Furutani M, Aida M (2006). *Arabidopsis CUP-SHAPED COTYLEDON3* regulates postembryonic shoot meristem and organ boundary formation. Plant Cell.

[63] Rojas-Murcia N, Hématy K, Lee Y, Emonet A, Ursache R, Fujita S (2020). High-order mutants reveal an essential requirement for peroxidases but not laccases in Casparian strip lignification. Proc Natl Acad Sci USA.

[64] Shukla V, Han J-P, Cléard F, Legendre-Lefebvre L, Gully K, Flis P (2021). Suberin plasticity to developmental and exogenous cues is regulated by a set of MYB transcription factors. Plant Biol.

[65] Ursache R, De Jesus Vieira Teixeira C, Dénervaud Tendon V, Gully K, De Bellis D, Schmid-Siegert E (2021). GDSL-domain proteins have key roles in suberin polymerization and degradation. Nat Plants.

[66] Pfister A, Barberon M, Alassimone J, Kalmbach L, Lee Y, Vermeer JE (2014). A receptor-like kinase mutant with absent endodermal diffusion barrier displays selective nutrient homeostasis defects. eLife.

[67] Doblas VG, Smakowska-Luzan E, Fujita S, Alassimone J, Barberon M, Madalinski M (2017). Root diffusion barrier control by a vasculature-derived peptide binding to the SGN3 receptor. Science.

[68] Fujita S, De Bellis D, Edel KH, Köster P, Andersen TG, Schmid-Siegert E (2020). SCHENGEN receptor module drives localized ROS production and lignification in plant roots. EMBO J.

[69] Wang X, Ye L, Lyu M, Ursache R, Löytynoja A, Mähönen AP (2020). An inducible genome editing system for plants. Nat Plants.

[70] Emonet A, Zhou F, Vacheron J, Heiman CM, Dénervaud Tendon V, Ma K-W (2021). Spatially restricted immune responses are required for maintaining root meristematic activity upon detection of bacteria. Curr Biol.

[71] Wilson P, Ganguly D, Hou X, Pogson B. CTAB genomic DNA extraction from *Arabidopsis *leaf material v2 (protocols.io.quidwue). https://www.protocols.io/view/ctab-genomic-dna-extraction-from-arabidopsis-leaf-quidwue. Accessed 19 July 2021.

[72] Ursache R, Andersen TG, Marhavý P, Geldner N (2018). A protocol for combining fluorescent proteins with histological stains for diverse cell wall components. Plant J.

