# Supplementary material for: Combined fluorescent seed selection and multiplex CRISPR/Cas9 assembly for fast generation of multiple Arabidopsis mutants
Source: Plant Methods. 2021 Oct 30;17:111. doi: 10.1186/s13007-021-00811-9 (PMC8556964; doi:10.1186/s13007-021-00811-9)
Supplement: Supplementary file 1 — Additional file 1: Table S1. 1st level intermediate vectors containing the corresponding BsaI for GoldenGate -based assembly. Table S2. 2nd level intermediate vectors. Please note that these vectors contain ccdB cassette and ccdB survival competent cells combined with Kanamycin and Chloramphenicol have to be used to propagate the empty vectors. Table S3. Primers for generation and modification of CRISPR vectors. Table S4. List of primers designed for gRNAs used in this study (overhangs for oligo annealing without leaving a scar after pU6 or pU3 promoters are highlighted in red). Table S5. Primers used for PCR-based genotyping. Table S6. List of the primers used for Cas9 amplification. [file 13007_2021_811_MOESM1_ESM.docx]

**Table S1.** 1^st^ level intermediate vectors containing the corresponding BsaI for GoldenGate -based assembly.

| **Vector** | **Addgene ID** | **Purpose** | **Bacterial selection** |
| --- | --- | --- | --- |
| pRU41 | 167663 | Entry vector for cloning gRNA1 driven by pU6 promoter | Ampicillin |
| pRU42 | 167664 | Entry vector for cloning gRNA2 driven by pU3 promoter | Ampicillin |
| pRU43 | 167665 | Entry vector for cloning gRNA3 driven by pU6 promoter | Ampicillin |
| pRU44 | 167666 | Entry vector for cloning gRNA4 driven by pU3 promoter | Ampicillin |
| pRU45 | 167667 | Entry vector for cloning gRNA5 driven by pU6 promoter | Ampicillin |
| pRU46 | 167668 | Entry vector for cloning gRNA6 driven by pU3 promoter | Ampicillin |
| pRU47 | 167669 | Entry vector for cloning gRNA7 driven by pU6 promoter | Ampicillin |
| pRU48 | 167670 | Entry vector for cloning gRNA8 driven by pU3 promoter | Ampicillin |

**Table S2**. 2^nd^ level intermediate vectors. Please note that these vectors contain ccdB cassette and ccdB survival competent cells combined with Kanamycin and Chloramphenicol have to be used to propagate the empty vectors.

| **Vector** | **Addgene ID** | **Gateway adapters** | **Purpose** | **Bacterial selection** |
| --- | --- | --- | --- | --- |
| pSF463 | 167671 | attL1-ccdB-attL2 | GoldenGate/Gateway vector for combining 2 gRNAs | Kanamycin |
| pSF464 | 167672 | attL1-ccdB-attL2 | GoldenGate/Gateway vector for combining 4 gRNAs | Kanamycin |
| pSF278 | 167673 | attL1-ccdB-attL2 | GoldenGate/Gateway vector for combining 3 gRNAs | Kanamycin |
| pSF279 | 167674 | attL1-ccdB-attL2 | GoldenGate/Gateway vector for combining 5 gRNAs | Kanamycin |
| pSF280 | 167675 | attL1-ccdB-attL2 | GoldenGate/Gateway vector for combining 6 gRNAs | Kanamycin |
| pRU325 | 167676 | attL1-ccdB-attL2 | GoldenGate/Gateway vector for combining 8 gRNAs | Kanamycin |

**Table S3.** Primers for generation and modification of CRISPR vectors

| Primer | Sequence | Purpose |
| --- | --- | --- |
| RU41.F | TGGTCGACCTGCAGGCGGCCGCGGTCTCGCTATCTTTTTTTCTTCTTCTTCG | pU6-gRNA1 cassette amplification for Infusion cloning |
| RU41.R | CCCGGCCGCCATGGCGGCCGGTCTCCCATGGCTGATCCTAAATGCTATC |  |
| RU42.F | ATGGTCGACCTGCAGGCGGCCGGTCTCCCATGCGTGTCTCAAAATCTCTGATGTTAC | pU3-gRNA2 cassette amplification for Infusion cloning |
| RU42.R | GGCCGCCATGGCGGCCGGTCTCGGTCCGCTGATCCTAAATGCTATCAAG |  |
| RU43.F | GTCGACCTGCAGGCGGCCGCGGTCTCAGGACCTTTTTTTCTTCTTCTTCGTTCAT | pU6-gRNA3 cassette amplification for Infusion cloning |
| RU43.R | GCCGCCATGGCGGCCGGTCTCCCTGGGCTGATCCTAAATGCTATCAAGTT |  |
| RU44.F | TGGTCGACCTGCAGGCGGCCGGTCTCGCCAGCGTGTCTCAAAATCTCTGATGTTAC | pU3-gRNA4 cassette amplification for Infusion cloning |
| RU44.R | GGCCGCCATGGCGGCCGGTCTCCAACAGCTGATCCTAAATGCTATCAAGT |  |
| RU45.F | TGGTCGACCTGCAGGCGGCCGGTCTCCTGTTCTTTTTTTCTTCTTCTTCGTTCAT | pU6-gRNA5 cassette amplification for Infusion cloning |
| RU45.R | CCCGGCCGCCATGGCGGCCGGTCTCCTGCAGCTGATCCTAAATGCTATCAA |  |
| RU46.F | TGGTCGACCTGCAGGCGGCCGGTCTCGTGCACGTGTCTCAAAATCTCTGATGTT | pU3-gRNA6 cassette amplification |
| RU46.R  RU47.F  RU47.R  RU48.F  RU48.R  SF278.F1  SF278.R1  SF278.F2  SF278.R2  SF278.F3  SF278.R3  SF278.F4  SF278.R4  SF279.F  SF279.R  SF280.F  SF280.R  SF463.F  SF463.R  SF464.F  SF464.R  RU325.F  RU325.R | CCCGGCCGCCATGGCGGCCGGTCTCACCGGCTGATCCTAAATGCTATCAAGTTTA  CCCATATGGTCGACCTGCAGGCGGCCGCGGTCTCGCGGTCTTTTTTTCTTCTTCTTCG  TCGCATGCTCCCGGCCGCCATGGCGGCCGGTCTCCTTTCGCTGATCCTAAATGCTATC  TCCCATATGGTCGACCTGCAGGCGGCCGGTCTCCGAA CGTCGCATGCTCCCGGCCGCCATGGCGGCCGGTCTCGTCGAGCTGATCCTAAATGCTAT  TTTGTACAAAAAAGCAGGCTTATACAAAAGTTGCCCCATGGCGTTCCCTC CTTTGTACAAGAAAGCTGGGTCGAATTCGCCCTTACTAGTCTGGAGAGACC  AGCCTGCTTTTTTGTACAAAGTTGGCA  ACCCAGCTTTCTTGTACAAAGTTGGC  AAGTGGTGGCTATCGAGACCGTCGACCGACAGCCTTCCAAATGTTCTTCTC  CTTACTAGTCTGGAGAGACCGTCGACTAAGTTGGCAGCATCACCCGACGC  GGTCTCTCCAGACTAGTAAGGGCGAATTCGACC  GGTCTCGATAGCCACCACTTGGGGATCCTG  TGCTGCCAACTTAGTCGACGGTCTCTTGCAACTAGTAAGGGCGAATTC  GAATTCGCCCTTACTAGTTGCAAGAGACCGTCGACTAAGTTGGCAGCA  GCCAACTTAGTCGACGGTCTCTCCGGTACTAGTAAGGGCGAATTC  GAATTCGCCCTTACTAGTACCGGAGAGACCGTCGACTAAGTTGGC  GCTGCCAACTTAGTCGACGGTCTCTGGACACTAGTAAGGGCGAATT  AATTCGCCCTTACTAGTGTCCAGAGACCGTCGACTAAGTTGGCAGC  GCTGCCAACTTAGTCGACGGTCTCTTGTTACTAGTAAGGGCGAATT  AATTCGCCCTTACTAGTAACAAGAGACCGTCGACTAAGTTGGCAGC  GCCAACTTAGTCGACGGTCTCTTCGAACTAGTAAGGGCGAATTCG  CGAATTCGCCCTTACTAGTTCGAAGAGACCGTCGACTAAGTTGGC | pU6-gRNA7 cassette amplification  pU3-gRNA8 cassette amplification  Amplification of BsaI- LacZ-BsaI from pYPQ143  Amplification of pDONR221 plasmid with recombined attL1-attL2 sites  ccdB amplification for replacing LacZ    pDONR221 amplification for ccdB insertion via Infusion  Site directed mutagenesis to change the BsaI sites for 5 gRNAs assembly  Site directed mutagenesis to change the BsaI sites for 5 gRNAs assembly  Site directed mutagenesis to change the BsaI sites for 2 gRNAs assembly  Site directed mutagenesis to change the BsaI sites for 4 gRNAs assembly  Site directed mutagenesis to change the BsaI sites for 8 gRNAs assembly |

**Table S4.** List of primers designed for gRNAs used in this study (overhangs for oligo annealing without leaving a scar after pU6 or pU3 promoters are highlighted in red)

| Target gene | gRNA | Forward primer | Reverse primer |
| --- | --- | --- | --- |
| *CUC1* | gRNA1 | ATTGATGTGTTTAACGGTTGGGGG | AAACCCCCCAACCGTTAAACACAT |
| *CUC1* | gRNA2 | GTCATGTTCGTTCTCAGTCCCGTT | AAACAACGGGACTGAGAACGAACA |
| *CUC1* | gRNA3 | ATTGGGACACGTGCTCCGTCGCAA | AAACTTGCGACGGAGCACGTGTCC |
| *CUC2* | gRNA1 | ATTGCCGTATTACCACTACGACCA | AAACTGGTCGTAGTGGTAATACGG |
| *CUC2* | gRNA2 | GTCAACCGGAGCCGTCTCCGAAGG | AAACCCTTCGGAGACGGCTCCGGT |
| *CUC2*  *SGN3*  *SGN3*  *SGN3* | gRNA3  gRNA1  gRNA2  gRNA3 | ATTGCGGTAGCCAGTAATTCATCC  ATTGGCTCAACG GGACGATACCGG  GTCAGTACAGTTGAACAGTTCAGT  ATTGGCACCAAACACTGAGTCGGT | AAACGGATGAATTACTGGCTACCG  AAACCCGGTATCGTCCCGTTGAGC  AAACACTGAACTGTTCAACTGTAC  AAACACCGACTCAGTGTTTGGTGC |

**Table S5.** Primers used for PCR-based genotyping

| Gene | Forward primer | Reverse primer |
| --- | --- | --- |
| *CUC1* | CACAGTCACGCACGCATTGCATGCTCAAAAGAC | TCCCAAATCCAGAAACTGACCAAACGCCACG |
| *CUC2*  *SGN3* | GGTATCTAGAAGCGACCGAAGAAATTCATT  CCGAGCCAGCTCGGCTCCCTCGTT AATATCCGGT | TCCACATTATTACCACGCCCCTTACTCAAG  CAGCTTCTTATAACCGGCCGTTCT GTTG |

**Table S6.** List of the primers used for Cas9 amplification

| Cas9 | Forward primer | Reverse primer |
| --- | --- | --- |
| spCas9 | TCGGAGAGTTGCACGCTATC | GGTAGGTTCCGAGAGATGCG |
| zCas9 | AGCATCCCACACCAGATTCA | CTCCTCGTTGTCCAGGAAGT |
