# Supplementary material for: Combined fluorescent seed selection and multiplex CRISPR/Cas9 assembly for fast generation of multiple Arabidopsis mutants
Source: Plant Methods. 2021 Oct 30;17:111. doi: 10.1186/s13007-021-00811-9 (PMC8556964; doi:10.1186/s13007-021-00811-9)
Supplement: Supplementary file 2 — Additional file 2: Figure S1. T-DNA vectors developed in this study. a Schematics showing the collection of final T-DNA vectors developed in this study. b Example of dominant presence of double-color fluorescent seeds in T2 generation. Scale bar = 100 μm. [file 13007_2021_811_MOESM2_ESM.pdf]

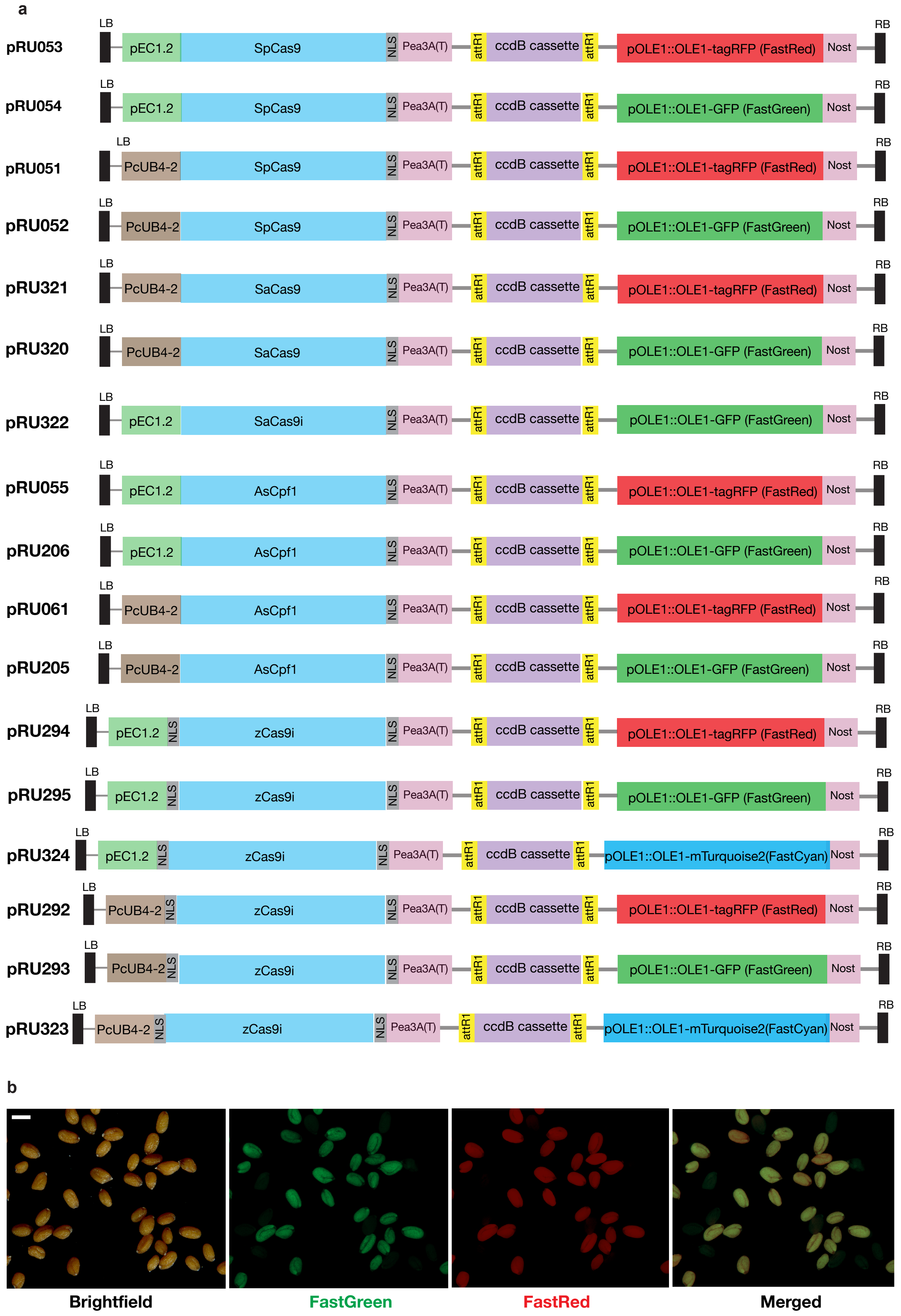

Figure S1. T-DNA vectors developed in this study. **a** Schematics showing the collection of final T-DNA vectors developed in this study. **b** Example of dominant presence of double-color fluorescent seeds in T2 generation. Scale bar = 100 μm.
