# Supplementary material for: Combined fluorescent seed selection and multiplex CRISPR/Cas9 assembly for fast generation of multiple Arabidopsis mutants
Source: Plant Methods. 2021 Oct 30;17:111. doi: 10.1186/s13007-021-00811-9 (PMC8556964; doi:10.1186/s13007-021-00811-9)
Supplement: Supplementary file 3 — Additional file 3: Materials and methods. CRISPR/Cas9 cloning protocol [file 13007_2021_811_MOESM3_ESM.docx]

**Supplementary Materials and Methods**

**CRISPR/Cas9 Cloning Protocol**

1. **Primer design**

Pick your 20 nucleotide protospacer sequence and order desalted oligos:

- **For pRU41 (pU6-gRNA1) vector:**

Forward primer: 5'-**ATTG** + protospacer

Reverse primer: 5'-**AAAC** + rev-com protospacer

- **For pRU42 (pU3-gRNA2) vector:**

Forward primer: 5'-**GTCA** + protospacer

Reverse primer: 5'-**AAAC** + rev-com protospacer

- **For pRU43 (pU6-gRNA3) vector:**

Forward primer: 5'-**ATTG** + protospacer

Reverse primer: 5'-**AAAC** + rev-com protospacer

- **For pRU44 (pU3-gRNA4) vector:**

Forward primer: 5'-**GTCA** + protospacer

Reverse primer: 5'-**AAAC** + rev-com protospacer

- **For pRU45 (pU6-gRNA5) vector:**

Forward primer: 5'-**ATTG** + protospacer

Reverse primer: 5'-**AAAC** + rev-com protospacer

- **For pRU46 (pU3-gRNA6) vector:**

Forward primer: 5'-**GTCA** + protospacer

Reverse primer: 5'-**AAAC** + rev-com protospacer

- **For pRU47 (pU6-gRNA7) vector:**

Forward primer: 5'-**ATTG** + protospacer

Reverse primer: 5'-**AAAC** + rev-com protospacer

- **For pRU48 (pU3-gRNA8) vector:**

Forward primer: 5'-**GTCA** + protospacer

Reverse primer: 5'-**AAAC** + rev-com protospacer

1. **Cloning gRNAs into pRU41-48 using Oligo annealing**

1 µl of each oligo (100 µM) + 48 µl H20

Incubate for 5 min at 95°C (thermocycler, no cooling at the end!)

Cooling at room temperature for 20 min

- **Digest entry vector:**

5 µl of corresponding pRU41-pRU48 entry vector
1 µl of FastDigest Buffer (Thermo Fisher Scientific)
1 µl FastDigest *BbsI* (*Bpi*) enzyme (Thermo Fisher Scientific, Catalog Nr. ER1011)
adjust water to 10 µl final volume and incubate for >1 h at 37°C (overnight is optimal)

Gel extract the digested vector and adjust the concentration to 5 ng/µl

- **Ligation**

2 µl of corresponding digested pRU41-48 entry vector
3 µl of annealed oligos
1,5 µl of T4 Ligase (Thermo Fisher Scientific, Catalog Nr. EL0011)
2 µl T4 buffer

1.5 µl H_2_O

Incubate for at least 1h at 22 °C or room temperature

Transform everything in chemically competent DH5α cells, plate on LB plates supplied with Ampicillin. We used the competent cells with the efficiency of 4.8 x 10^8^ transformants/µg plasmid DNA.

- **Colony-PCR**

Test 4 colonies (efficiency >70%) using oRU385 + gRNA reverse oligo

- **Miniprep**

Sequence using oRU385 primer and adjust plasmid concentrations to 100 ng/µl

1. **Golden Gate Assembly:**

**For two gRNAs:**

T4 DNA ligase buffer (Thermo Fisher) - 1 µl

FastDigest buffer (Thermo Fisher) – 1 µl

**pRU41** (pU6-gRNA1) – 1 µl

**pRU42** (pU3-gRNA2) – 1 µl

**pSF463** Intermediate vector – 1 µl

*Eco31I (BsaI)* (Thermo Fisher Scientific, Catalog. Nr. ER0291)– 1.5 µl

T4 ligase – 1.5 µl

H_2_O – 2.0 µl

**For three gRNAs:**

T4 DNA ligase buffer (Thermo Fisher) - 1 µl

FastDigest buffer (Thermo Fisher) – 1 µl

**pRU41** (pU6-gRNA1) – 1 µl

**pRU42** (pU3-gRNA2) – 1 µl

**pRU43** (pU6-gRNA3) – 1 µl

**pSF278** Intermediate vector– 1 µl

*Eco31I (BsaI)* (Thermo Fisher) – 1.5 µl

T4 ligase – 1.5 µl

H_2_O – 1.0 µl

**For four gRNAs:**

T4 DNA ligase buffer (Thermo Fisher) - 1 µl

FastDigest buffer (Thermo Fisher) – 1 µl

**pRU41** (pU6-gRNA1) – 1 µl

**pRU42** (pU3-gRNA2) – 1 µl

**pRU43** (pU6-gRNA3) – 1 µl

**pRU44** (pU3-gRNA4) – 1 µl

**pSF464** Intermediate vector – 1 µl

*Eco31I (BsaI)* (Thermo Fisher) – 1.5 µl

T4 ligase (Thermo Fisher) – 1.5 µl

H_2_O – 1.0 µl

**For five gRNAs:**

T4 DNA ligase buffer (Thermo Fisher) - 1 µl

FastDigest buffer (Thermo Fisher) – 1 µl

**pRU41** (pU6-gRNA1) – 1 µl

**pRU42** (pU3-gRNA2) – 1 µl

**pRU43** (pU6-gRNA3) – 1 µl

**pRU44** (pU3-gRNA4) – 1 µl

**pRU45** (pU6-gRNA5) – 1 µl

**pSF279** Intermediate vector – 1 µl

*Eco31I (BsaI)* (Thermo Fisher) – 1.5 µl

T4 ligase (Thermo Fisher) – 1.5 µl

H_2_O – 1.0 µl

**For eight gRNAs:**

T4 DNA ligase buffer (Thermo Fisher) - 1 µl

FastDigest buffer (Thermo Fisher) – 1 µl

**pRU41** (pU6-gRNA1) – 1 µl

**pRU42** (pU3-gRNA2) – 1 µl

**pRU43** (pU6-gRNA3) – 1 µl

**pRU44** (pU3-gRNA4) – 1 µl

**pRU45** (pU6-gRNA5) – 1 µl

**pRU46** (pU3-gRNA6) – 1 µl

**pRU47** (pU6-gRNA7) – 1 µl

**pRU48** (pU3-gRNA8) – 1 µl

**pRU325** Intermediate vector – 1 µl

*Eco31I (BsaI)* (Thermo Fisher) – 1.5 µl

T4 ligase (Thermo Fisher) – 1.5 µl

H_2_O – 1.0 µl

**For six gRNAs:**

T4 DNA ligase buffer (Thermo Fisher) - 1 µl

FastDigest buffer (Thermo Fisher) – 1 µl

**pRU41** (pU6-gRNA1) – 1 µl

**pRU42** (pU3-gRNA2) – 1 µl

**pRU43** (pU6-gRNA3) – 1 µl

**pRU44** (pU3-gRNA4) – 1 µl

**pRU45** (pU6-gRNA5) – 1 µl

**pRU46** (pU3-gRNA6) – 1 µl

**pSF280** Intermediate vector – 1 µl

*Eco31I (BsaI)* (Thermo Fisher) – 1.5 µl

T4 ligase (Thermo Fisher) – 1.5 µl

H_2_O – 1.0 µl

**Run Golden Gate program in a thermocycler as follows:**

37 ºC, 5 min

20 cycles

16 ºC, 10min

50 ºC, 5 min,

80 ºC, 5 min

Hold at 10 ºC

- Transform everything into E.coli DH5α cells and plate transformed cells on LB/Kan plates.
- Check 2-4 colonies, miniprep, sequence using M13 and M13 reverse primers. To check the gRNA’s in the middle, gRNA primers can be used as colony PCR and sequencing primers.

1. **Final Single Fragment Gateway LR Reaction**

2 µl of your intermediate vector with gRNAs assembled (adjusted to 50 ng/µl)
3 µl of the final Cas9 vector (adjusted to 50 ng/µl)
4 µl TE buffer, pH 8
1 µl LR clonase II enzyme mix (Thermo Fisher Scientific, Catalog Nr. 11791020)

- Incubate overnight at room temperature
- Proteinase K treatment: add 1 µl and incubate for 10 min at 37°C
- Transform everything in DH5α and plate everything on LB plates with Spectinomycin
- Miniprep, all colonies should be positive, inoculate 1-2 colonies
- Sequence using oRU906, oRU908 primers or gRNAs as primers

**Primer Sequences**

oRU906: GAGTCTATGATCAAGTAATTATGC

oRU908: GCTTGCATGCCTGCAGGTCGACTCT

oRU385: CAACGCGTTGGGAGCTCTCCCATATG
